# Factors Associated with Myelosuppression Related to Low-Dose Methotrexate Therapy for Inflammatory Rheumatic Diseases

**DOI:** 10.1371/journal.pone.0154744

**Published:** 2016-04-29

**Authors:** Shunsuke Mori, Michihiro Hidaka, Toshiro Kawakita, Toshihiko Hidaka, Hiroyuki Tsuda, Tamami Yoshitama, Kiyoshi Migita, Yukitaka Ueki

**Affiliations:** 1 Department of Rheumatology, Clinical Research Center for Rheumatic Diseases, NHO Kumamoto Saishunsou National Hospital, Kohshi, Kumamoto 861–1196, Japan; 2 Department of Hematology, NHO National Kumamoto Medical Center, Kumamoto 860–0008, Japan; 3 Institute of Rheumatology, Zenjinkai Shimin-no-Mori Hospital, Miyazaki 880–0122, Japan; 4 Department of Hematology and Oncology, Kumamoto City Hospital, Kumamoto 862–8505, Japan; 5 Yoshitama Clinic for Rheumatic Diseases, Kirishima, Kagoshima 899–5117, Japan; 6 Clinical Research Center, NHO National Nagasaki Medical Center, Omura, Nagasaki 856–8652, Japan; 7 Rheumatic and Collagen Disease Center, Sasebo Chuo Hospital, Sasebo, Nagasaki 857–1195, Japan; Nippon Medical School Graduate School of Medicine, JAPAN

## Abstract

**Objective:**

Severe myelosuppression is a serious concern in the management of rheumatic disease patients receiving methotrexate (MTX) therapy. This study was intended to explore factors associated with the development of MTX-related myelosuppression and its disease severity.

**Methods:**

We retrospectively examined a total of 40 cases of MTX-related myelosuppression that had been filed in the registries of participating rheumatology and hematology divisions. Data before onset were compared with those of 120 controls matched for age and sex. Cytopenia was graded according to the National Cancer Institute criteria for adverse events. Data before and at onset were compared between the severe and non-severe groups.

**Results:**

Non-use of folic acid supplements, concurrent medications, and low renal function were significantly associated with the development of myelosuppression (*p* < 0.001, *p* < 0.001, and *p* = 0.002, respectively). In addition, significantly lower MTX dosages, higher blood cell counts, and lower hemoglobin levels were seen in the myelosuppression group (*p* < 0.001). No patients exhibited leukocytopenia, neutropenia, or thrombocytopenia in routine blood monitoring taken within the past month. One-fourth developed myelosuppression within the first two months (an early-onset period). Myelosuppression was severe in approximately 40% of patients. Hypoalbuminemia and non-use of folic acid supplements were significantly associated with the severity of pancytopenia (*p* = 0.001 and 0.008, respectively). Besides these two factors, early onset and the use of lower doses of MTX were significantly associated with the severity of neutropenia (*p* = 0.003, 0.007, 0.003, and 0.002, respectively).

**Conclusions:**

Myelosuppression can occur abruptly at any time during low-dose MTX therapy, but severe neutropenia is more likely to occur in the early-onset period of this therapy. Contrary to our expectations, disease severity was not dependent on MTX doses. Serum albumin levels and folic acid supplementation are the important factors affecting the severity of MTX-related pancytopenia and neutropenia.

## Introduction

Methotrexate (MTX) is widely recognized as a key conventional synthetic disease-modifying antirheumatic drug (DMARD) in the treatment of inflammatory rheumatic diseases, especially rheumatoid arthritis (RA). This drug continues to serve as an anchor drug among DMARDs, mainly based on the following characteristics: its proven efficacy both during monotherapy and in combination with steroids, other conventional systemic DMARDs, and biological DMARDs; the ability to increase the efficacy of biological DMARDs when used in combination; and the acceptable long-term safety profile [1–3]. The 2013 update of EULAR recommendations for the management of RA with synthetic and biological DMARDs did not change the previous recommendation that MTX should be part of the first strategy in patients with active RA [4].

Although MTX was originally developed for use in anti-cancer therapy as part of a high-dose regimen, low-dose MTX has proven to be highly effective in the treatment of RA. Multinational evidence-based recommendations state that oral MTX be started at 10 to 15 mg/week, with an escalation of 5 mg every two to four weeks up to 20 to 30 mg/week, depending on clinical response and tolerability [5]. In Japan, an MTX dosage of up to 16 mg/week is currently approved. Low-dose MTX therapy is usually well tolerated in clinical practice, and most adverse events are moderate and resolve following drug discontinuation [6–9]. However, long-term clinical experience with MTX in the treatment of RA has taught us that MTX can induce severe, potentially life-threatening adverse events, especially opportunistic infections (*Pneumocystis jirovecii* pneumonia, mycobacterial infections, and viral reactivation such as *de novo* hepatitis B and disseminated herpes zoster infection, etc.), pulmonary toxicity, and myelosuppression [10–13].

MTX-related myelosuppression is estimated to occur in 2 to 10.2% of patients with inflammatory rheumatic diseases [14–17]. Neutropenia is encountered most often, but anemia and thrombocytopenia also occur [14, 16–18]. Neutropenia and pancytopenia developed in 1.4 to 7% and 0.3 to 2.1% of patients receiving low-doses of MTX, respectively [15–23]. Although severe or fatal cases of myelosuppression have been reported in this clinical context, it is not clear which factors contribute to disease severity. It is generally believed that the severity of acute adverse effects is related to dose and frequency of MTX administration; however, a recent study suggested that serum MTX concentrations did not correlate with the degree of either neutropenia or thrombocytopenia [24].

For a better understanding of possible factors associated with the development of MTX-related myelosuppression and its disease severity in daily practice for inflammatory rheumatic diseases, we collected data from all cases that had been filed in the registries of participating rheumatology and hematology divisions and compared clinical and laboratory features between the cases and age- and sex-matched controls as well as between patient groups after stratification according to the severity of pancytopenia or neutropenia. The contribution of individual factors was discussed in the context of pathogenesis of MTX-related myelosuppression.

## Patients and Methods

### Study design and patients

The following divisions for rheumatology or hematology registered all patients who were treated for hematological disorders, including myelosuppression, because they are required to prepare and disclose annual reports regarding exact numbers of cases for individual diseases: Department of Rheumatology, NHO Kumamoto Saishunsou National Hospital; Department of Hematology, NHO National Kumamoto Medical Center; Institute of Rheumatology, Zenjinkai Shimin-no-Mori Hospital; Department of Hematology and Oncology, Kumamoto City Hospital; Yoshitama Clinic for Rheumatic Diseases; Clinical Research Center, NHO National Nagasaki Medical Center; and Rheumatic and Collagen Disease Center, Sasebo Chuo Hospital. By using the terms of myelosuppression, MTX, and rheumatic diseases, we searched these registry data and captured patients (of 18 years and over) who had been treated for myelosuppression related to MTX therapy for rheumatic diseases, and we then identified and enrolled eligible patients who fulfilled the following inclusion and exclusion criteria for this study. Myelosuppression was defined as at least one of the following conditions: leukopenia (leukocyte count below 3500/mm^3^), thrombocytopenia (platelet count below 130000/mm^3^), or anemia (hemoglobin level less than 11.5 g/dl for females or 13.5 g/dl for males). Since it is well known that patients with chronic inflammatory diseases such as RA often suffer from anemia [25, 26], we included a decrease of 0.5 g/dl or more than the latest hemoglobin values measured within one month to the definition criteria for newly developed anemia during MTX therapy. Patients with other possible causes known to be associated with myelosuppression were excluded from this study. As for the age- and sex-matched controls, we selected 120 rheumatic disease patients who had received MTX therapy in the participating divisions but had not developed myelosuppression.

From among all eligible patients, we identified patients who were diagnosed with pancytopenia, defined as the presence of leukopenia, thrombocytopenia, and anemia. Severe pancytopenia was defined as a leukocyte count below 2000/mm^3^, a platelet count below 50000/mm^3^, and a hemoglobin level less than 10.0 g/dl [22]. We also identified patients who were diagnosed with neutropenia, which was defined as a neutrophil count below 2000/mm^3^. Severe neutropenia (agranulocytosis) was defined as a decrease in a neutrophil count to less than 500/mm^3^ [27]. Patients’ cytopenia and hypoalbuminemia were graded according to the severity grade of the National Cancer Institute Common Terminology Criteria for Adverse Events (NCI-CTCAE) version 4.0 (http://ctep.cancer.gov).

Through reviewing patients’ medical records, we scrutinized demographic data, laboratory results before and at onset of myelosuppression, indications for MTX therapy, MTX dosages and duration, medications concurrently used during MTX therapy, preceding episodes of dehydration, folic acid supplementation, and clinical presentation at onset. The data were compared between the severe and non-sever patient groups.

This study was conducted in accordance with the principles of the Declaration of Helsinki (2008). The protocol of this study also meets the requirements of the Ethical Guidelines for Medical and Health Research Involving Human Subjects, Japan (2014) and has been approved by the Human Research Ethics Committees of NHO Kumamoto Saishunsou National Hospital. Since the data were analyzed anonymously, patient consent was not required. This study is registered with the University Hospital Medical Information Network-Clinical Trials Registry (UMIN000020906).

### Renal function

An estimated glomerular filtration rate (eGFR) was first calculated according to the following equation that had been developed for Japanese patients and was officially approved by the Japanese Society of Nephrology: eGFR (ml/min/1.73 m^2^) = 194 × (serum creatinine [mg/dl])^-1.094^ × (age)^-0.287^ × 0.739 (if female) [28] and was then corrected for each patient’s BSA. Corrected eGFR = GFR × BSA / 1.73. Renal insufficiency was defined as a corrected eGFR < 60 ml/min. eGFR categories were determined according to the Kidney Disease Improving Global Outcomes (KDIGO 2012) guidelines.

### Statistical analysis

Statistical analyses were performed using the independent-measures t-test for comparisons of continuous variables between two groups. The chi-square test using 2 ×2 contingency tables was used for categorical variables. If cell values were less than 5, Fisher’s exact probability test was used. The Pearson’s correlation technique was used to investigate the degree of the relationship between weekly MTX doses prescribed and corrected eGFR values before onset of myelosuppression. For all tests, probability values (*p* values) < 0.01 were considered to indicate statistical significance. All calculations were performed using PASW Statistics version 22 (SPSS Japan Inc., Tokyo, Japan).

## Results

### Clinical and laboratory features of patients who developed myelosuppression during low-dose MTX therapy

Through searching the registries of the participating divisions, we identified a total of 40 patients who had developed myelosuppression during low-dose MTX therapy for inflammatory rheumatic disease between February 2005 and November 2014 (Table 1). Thirty-nine cases occurred in patients with RA and one in a patient who had been diagnosed with polymyalgia rheumatica (PMR). More than 90% of patients were 65 years of age or more. When compared with the age- and sex-matched control group, rates of folic acid supplementation were significantly lower in patients who had developed myelosuppression (cases versus controls: 42.5% versus 100%, *p* < 0.001). Corrected eGFR values before onset were also significantly lower in the case group (46.7 ml/min versus 56.3 ml/min, *p* = 0.002). In the case group, more patients had received concomitant medications (97.5% versus 62.5%, *p* < 0.001), especially nonsteroidal anti-inflammatory drugs (NSAIDS, 72.5% versus 10%, *p* < 0.001), antacids (45% versus 15%, *p* < 0.001), and prednisolone (67.5% versus 12.5%, *p* < 0.001). Counts of blood cells, such as leukocytes, neutrophils, and thrombocytes, were significantly higher in the case group (*p* < 0.001). Hemoglobin levels were lower in the case group (10.6 mg/dl versus 12.3 mg/dl, *p* < 0.001). Weekly MTX doses were significantly lower in the case group (5.7 mg versus 7.3 mg, *p* < 0.001). There was a significant correlation between MTX doses prescribed and corrected eGFR values before onset in those patients who had received MTX therapy (r = 0.421, *p* < 0.001).

**Table 1:** Clinical and laboratory characteristics of patients who developed myelosuppression during low-dose MTX.

	Case (n = 40)	Control^§^ (n = 120)	*p*
Age, years, mean (SD)	73.0 (8.7)	-	-
> 65, patient number (%)	37 (92.5)	-	-
Female/male, patient number	34/6	-	-
Indication for MTX therapy, RA/PMR, patient number	39/1	120/0	-
MTX therapy			
Doses, mg/week, mean (SD)	5.7 (2.0)	7.3 (2.1)	<0.001
Treatment duration, months, mean (SD)	44.5 (48.9)	68.7 (49.2)	0.008
Early onset*, patient number (%)	11 (27.5)	-	-
Folic acid supplementation, patient number (%)	17 (42.5)	120 (100)	<0.001
Concurrent medications, patient number (%)	39 (97.5)	75 (62.5)	<0.001
NSAIDs	29 (72.5)	12 (10)	<0.001
Other biological and systemic DMARDs	12 (30)^†^	32 (26.7)	0.68
Antiarrhythmic drugs	3 (7.5)	3 (2.5)	0.17
ARB/ACE inhibitors	7 (17.5)	33 (27.5)	0.29
Antacids	18 (45)	18 (15)	<0.001
H2 blockers	6 (15)	1 (0.8)	0.001
Proton pump inhibitors	12 (30)	17 (14.2)	0.033
Prednisolone	27 (67.5)	15 (12.5)	<0.001
None	1 (2.5)	45 (37.5)	<0.001
BMI, kg/mm^2^, mean (SD)	20.3 (2.5)	24.1 (16.1)	0.14
Preceding episodes of dehydration, patient number (%)	18 (45)	-	-
Laboratory data before onset^‡^			
Leucocytes, /mm^3^, mean (SD)	6741 (2324)	5003 (1445)	<0.001
Neutrophils, /mm^3^, mean (SD)	4696 (2378)	3264 (1378)	<0.001
Thrombocytes, x 10^4^/mm^3^, mean (SD)	25.3 (9.9)	18.6 (4.8)	<0.001
Hemoglobin, g/dl, mean (SD)	10.6 (1.4)	12.3 (1.6)	<0.001
Corrected eGFR, ml/min, mean (SD)	46.7 (21.6)	56.3 (15.5)	0.002

*Defined as the development of pancytopenia within the first two months of MTX therapy.

^†^Other DMARDs included salazosulfapyridine (n = 8), tumor necrosis factor inhibitors (n = 3), and tocilizumab (n = 1).

^‡^The latest available data within one month before onset of myelosuppression.

^§^Controls were selected by individual matching for age and sex. MTX, methotrexate; RA, rheumatoid arthritis; PMR polymyalgia rheumatica; eGFR, estimated glomerular filtration rate; NSAIDs, nonsteroidal anti-inflammatory drugs; DMARDs, disease-modifying antirheumatic drugs; ARB, angiotensin receptor blockers; ACE, angiotensin converting enzyme; BMI, body mass index; SD, standard deviation.

Eleven patients (27.5%) developed myelosuppression within the first two months of MTX therapy. Eighteen patients (45%) had a preceding episode that could have caused dehydration (anorexia, seven cases; stomatitis, four cases; flu, three cases; pyelonephritis, two cases; pharyngitis, one case; diarrhea, one case). The onset was abrupt: no patients exhibited leukocytopenia, neutropenia, or thrombocytopenia in routine blood monitoring taken within one month before onset.

### Severity of myelosuppression related to low-dose MTX therapy for inflammatory rheumatic diseases

As shown in Table 2, among the 40 cases of myelosuppression related to low-dose MTX therapy, 31 cases (77.5%) were pancytopenia, 8 (20%) were bicytopenia (three cases, leukopenia and thrombocytopenia; three cases, leukopenia and anemia; two cases, thrombocytopenia and anemia), and 1 (2.5%) was isolated neutropenia. Severe pancytopenia was observed in 12 out of 31 patients (38.7%). Patients with a severity grade of 4 accounted for 39.5% of leukopenia, 38.9% of thrombocytopenia, and 27.8% of anemia cases. Thirty-seven patients (92.5%) developed neutropenia, and among these, 16 (43.2%) were diagnosed with severe neutropenia (corresponding to a severity grade of 4). In nine patients, neutrophil counts were below 100/mm^3^, and among those, five were found to have neutrophil counts of zero. Hypoalbuminemia and renal insufficiency were observed in the majority of myelosuppression patients (95% and 90%, respectively). Approximately half of the patients experienced a rapid exacerbation of renal function. Five patients had hepatotoxicity at the onset of myelosuppression, but no case progressed to liver failure. No patient showed pulmonary toxicity, namely interstitial pneumonia. Half of the cases were complicated by infectious diseases, and among these, 11 developed sepsis.

**Table 2:** Severity of myelosuppression related to low-dose MTX therapy for inflammatory rheumatic diseases (n = 40).

Type of myelosuppression	
Pancytopenia, patient number (%)	31 (77.5)
Severe pancytopenia, patient number (%)	12 (38.7)
Bicytopenia, patient number (%)	8 (20)
Isolated leukopenia, patient number (%)	1 (2.5)
Leucocytes, /mm^3^, mean (SD)	1757 (1424)
Leucopenia, patient number (%)	38 (95)
Grade 1 (≥3000 and <3500)	3 (7.9)
Grade 2 (≥2000 and <3000)	13 (34.2)
Grade 3 (≥1000 and <2000)	7 (18.4)
Grade 4 (<1000)	15 (39.5)
Neutrophils, /mm^3^, mean (SD)	972 (1272)
Neutropenia, patient number (%)	37 (92.5)
Grade 1 (≥1500 and <2000)	3 (8.1)
Grade 2 (≥1000 and <15000)	7 (18.9)
Grade 3 (≥500 and <1000)	11 (29.7)
Grade 4 (<500)	16 (43.2)
Thrombocytes, /mm^3^, mean (SD)	6.3x 10^4^ (5.5 x 10^4^)
Thrombocytopenia, patient number (%)	36 (90)
Grade 1 (≥75000 and <130000)	9 (25)
Grade 2 (≥50000 and <75000)	8 (22.2)
Grade 3 (≥25000 and <50000)	5 (13.9)
Grade 4 (<25000)	14 (38.9)
Hemoglobin, g/dl, mean (SD)	7.8 (2.4)
Anemia, patient number (%)	36 (90)
Grade 1 (≥10.0 and <11.5 (for female) or 13.5 (for male)	5 (13.9)
Grade 2 (≥8.0 and <10.0)	9 (25)
Grade 3 (≥6.5 and <8.0)	12 (33.3)
Grade 4 (<6.5)	10 (27.8)
Albumin, g/dl, mean (SD)	2.9 (0.7)
Hypoalbuminemia, patient number (%)	38 (95)
Grade 1 (≥3.0 and <4.0)	17 (44.7)
Grade 2 (≥2.0 and <3.0)	17 (44.7)
Grade 3 (<2.0)	4 (10.5)
Corrected eGFR, ml/min, mean (SD)	35.1 (21.1)
eGFR categories	
G1 (≥90)	1 (2.5)
G2 (≥60 and <90)	3 (7.5)
G3 (≥30 and <60)	19 (47.5)
G4 (≥15 and <30)	9 (22.5)
G5 (<15)	8 (20)
Rapid exacerbation of renal function*, patient number (%)	21 (52.5)
Hepatotoxicity^†^, patient number (%)	5 (12.5)
Mucositis at onset, patient number (%)	18 (45)
Pulmonary toxicity (interstitial pneumonia) (%)	0
Infectious diseases^‡^, patient number (%)	20 (50)
Sepsis, patient number (%)	11 (27.5)
Outcomes, died/recovered, patient number	5/35

*Defined as an increase of 0.3 mg/dl or more or a twofold increase or more in serum creatinine levels compared with the latest available data within one month before the development of myelosuppression.

^†^Defined as an at least twofold elevation in serum alanine aminotransferase and/or aspartate aminotransferase levels over the upper limit of normal. No patients developed liver failure.

^‡^Infectious diseases include pneumonia (n = 10), pyelonephritis (n = 3), enteritis (n = 2), and stomatitis caused by EB virus (n = 1), and sepsis from unknown focuses (n = 3). MTX, methotrexate; eGFR, estimated glomerular filtration rate; SD, standard deviation.

Three patients were under hemodialysis. All of the patients developed s severity grade of 4 for neutropenia and thrombocytopenia within one month. Weekly MTX doses prescribed were very small, ranging from 2 to 4 mg. After the introduction of MTX therapy, their renal function rapidly exacerbated.

Despite intensive treatment with intravenous antibiotics, leucovorin, granulocyte colony stimulating factor, and transfusions of blood products including packed red cell, platelets, and fresh frozen plasma, five patients died of sepsis. Among the fatal cases, four were severe pancytopenia with grade 4 neutropenia and thrombocytopenia. One case was bicytopenia with grade 3 anemia and grade 4 thrombocytopenia. This patient’s disease was complicated by disseminated intravascular coagulation. Two patients had received only a total of 4–6 mg of MTX (case 1, two doses of 2 mg; case 2, a single dose of 6 mg). One case (case 1) occurred in a patient under hemodialysis.

### Factors affecting the severity of pancytopenia and neutropenia

As shown in Table 3, non-use of folic acid supplements and serum albumin levels were significantly associated with the severity of pancytopenia (non-severe versus severe: 57.9% versus 8.3%, *p* = 0.008; 3.1 g/dl versus 2.3 g/dl, *p* = 0.001, respectively). There were no differences in MTX doses prescribed for both groups. Patients with severe pancytopenia were more likely to develop infectious diseases (31.6% versus 83.3%, *p* = 0.009) and sepsis (5.3% versus 75%, *p* < 0.001). Fatal outcomes were seen only in patients with severe pancytopenia.

**Table 3:** Factors associated with severity of MTX-related pancytopenia (n = 31).

	Non-severe (n = 19)	Severe (n = 12)	*p*
Age, years, mean (SD)	73.8 (8.1)	71.9 (5.8)	0.48
Female/male, patient number	15/4	10/2	1.00
MTX doses, mg/week, mean (SD)	6.3 (2.1)	4.8 (1.8)	0.053
Duration of MTX therapy, months, mean (SD)	45.3 (46.5)	34.8 (39.1)	0.52
Early onset*, patient number (%)	4 (21.1)	4 (33.3)	0.68
Folic acid supplementation, patient number (%)	11 (57.9)	1 (8.3)	0.008
Concurrent medications, patient number (%)	18 (94.7)	11 (91.7)	1.00
BMI, kg/m^2^, mean (SD)	20.5 (2.3)	19.4 (3.0)	0.27
Preceding dehydration, patient number (%)	8 (44.4)	6 (50)	0.67
Albumin at onset, g/dl, mean (SD)	3.1 (0.6)	2.3 (0.5)	0.001
<3.0 g/dl at onset, patient number (%)	8 (47.1)	11 (91.7)	0.008
Corrected eGFR, ml/min, mean (SD)			
Before onset	45.4 (13.3)	43.2 (29.9)	0.78
At onset	38.6 (17.9)	23.8 (16.0)	0.027
Rapid exacerbation of renal function^†^, patient number (%)	9 (47.4)	9 (75)	0.16
Hepatotoxicity at onset^‡^, patient number (%)	3 (15.8)	2 (16.7)	1.00
Mucositis at onset, patient number (%)	7 (36.8)	7 (58.3)	0.24
Infectious diseases at onset, patient number (%)	6 (31.6)	10 (83.3)	0.009
Complicated sepsis, patient number (%)	1 (5.3)	9 (75)	<0.001
Fatal outcome, patient number (%)	0	4 (33.3)	0.016

*Defined as development of pancytopenia within the first two months of MTX therapy.

^†^Defined as an increase of 0.3 mg/dl or more or a twofold increase or more in serum creatinine levels compared with the latest available data within one month before onset of pancytopenia.

^‡^Defined as an at least twofold elevation in serum alanine aminotransferase and aspartate aminotransferase levels over the upper limit of normal. MTX, methotrexate; BMI, body mass index; eGFR, estimated glomerular filtration rate; SD, standard deviation.

We also compared the data between patients who had developed severe neutropenia and those with non-severe neutropenia (Table 4). Like severe pancytopenia, non-use of supplemental folic acid and serum albumin levels were the significant factors affecting the disease severity (non-severe versus severe: 66.7% versus 18.8%, *p* = 0.007; 3.2 g/dl versus 2.5 g/dl, *p* = 0.003, respectively). In addition, severe neutropenia was more likely to occur within the first two months than was non-severe neutropenia (9.5% versus 56.3%, *p* = 0.003). MTX doses were significantly lower in severe neutropenia patients than in non-severe neutropenia patients (6.6 mg/week versus 4.6 mg/week, *p* = 0.002). Complication of infectious diseases and sepsis were more often seen in severe neutropenia patients than in non-severe neutropenia patients (33.3% versus 75%, *p* = 0.02; 9.5% versus 50%, *p* = 0.009, respectively). All of the fatal cases were seen in severe neutropenia patients.

**Table 4:** Factors associated with severity of MTX-related neutropenia (n = 37).

	Non-severe (n = 21)	Severe (n = 16)	*p*
Age, years, mean (SD)	73.4 (8.9)	70.8 (8.3)	0.37
Female/male, patient number	17/4	14/2	0.68
MTX doses, mg/week, mean (SD)	6.6 (1.9)	4.6 (1.7)	0.002
Duration of MTX therapy, months, mean (SD)	58.8 (53.9)	25.0 (37.6)	0.039
Early onset*, patient number (%)	2 (9.5)	9 (56.3)	0.003
Folic acid supplementation, patient number (%)	14 (66.7)	3 (18.8)	0.007
Concurrent medications, patient number (%)	20 (95.2)	15 (93.8)	1.00
BMI, kg/m^2^, mean (SD)	20.5 (2.3)	19.9 (2.7)	0.44
Preceding dehydration, patient number (%)	7 (33.3)	10 (62.5)	0.078
Albumin at onset g/dl, mean (SD)	3.2 (0.6)	2.5 (0.7)	0.003
<3.0 g/dl at onset, patient number (%)	7 (33.3)	13 (81.3)	0.007
Corrected eGFR, ml/min, mean (SD)			
Before onset	48.6 (14.3)	42.6 (30.0)	0.46
At onset	39.7 (21.3)	29.3 (21.3)	0.15
Rapid exacerbation of renal function^†^, patient number (%)	8 (38.1)	11 (68.8)	0.065
Hepatotoxicity at onset^‡^, patient number (%)	3 (14.3)	2 (12.5)	1.00
Mucositis at onset, patient number (%)	6 (28.6)	10 (62.5)	0.039
Infectious diseases at onset, patient number (%)	7 (33.3)	12 (75)	0.020
Complicated sepsis, patient number (%)	2 (9.5)	8 (50)	0.009
Fatal outcome, patient number (%)	0	4 (25)	0.028

*Defined as development of neutropenia within the first two months of MTX therapy.

^†^Defined as an increase of 0.3 mg/dl or more or a twofold increase or more in serum creatinine levels compared with the latest available data within one month before onset of neutropenia.

^‡^Defined as an at least twofold elevation in serum alanine aminotransferase and aspartate aminotransferase levels over the upper limit of normal. MTX, methotrexate; BMI, body mass index; eGFR, estimated glomerular filtration rate; SD, standard deviation.

## Discussion

In the present study, serum albumin levels were identified as the significant factor that affected the severity of pancytopenia and neutropenia. Hypoalbuminemia has been reported as the factor predisposing rheumatic disease patients to myelosuppression [20–22, 24, 29–31]. MTX is moderately bound to plasma proteins, mainly albumin, with the fraction bound ranging from 46.5 to 54% [32]. MTX unbound to albumin, but not the bound form, can enter cells via the reduced folate carrier, and then MTX is converted to polyglutamate derivatives, which enhances its intracellular retention [33]. Since the toxic effects of MTX are considered to depend on the intracellular drug concentration and exposure duration, the increase in extracellular concentrations of unbound MTX can contribute to the development of severe myelosuppression. The reduction of serum albumin levels would lead to an increase in unbound MTX. Although hepatic impairment is known to decrease albumin production, only 12.5% of patients in the present study showed hepatotoxicity at the time of development of myelosuppression, and none developed liver failure. The chronic nature of RA may be involved in hypoalbuminemia seen in our patient population.

Myelosuppression occurs more commonly as a late complication of low-dose MTX therapy, but it can also occur in an early-onset period (within one to two months), independently of dosage, possibly as an idiosyncratic reaction to MTX. MTX-related myelosuppression is generally considered to result from toxicity due to drug effects on folate antagonism in bone marrow, but the idiosyncratic reaction appears to be an immunological or hypersensitive phenomenon, which is unpredictable and can result in significant morbidity and mortality [34–36]. In the present study, one-fourth of patients developed myelosuppression within the first two months. MTX doses were much lower in those patients who had developed myelosuppression. In particular, severe neutropenia was more frequently seen in patients treated with lower doses as well as during the early-onset period of MTX therapy. However, we cannot exclude the possibility that the association between the use of lower MTX doses and the development of myelosuppression was simply due to the therapeutic selection of using lower doses of MTX for patients with worse renal function, because there was a significant correlation between corrected eGFR values and MTX doses. In both of the early-onset and late-onset cases, the occurrence of myelosuppression was abrupt, and it was difficult to detect this complication in its early stages, even though monthly blood monitoring was performed.

MTX and its metabolites are primarily eliminated in the urine through glomerular filtration and active tubular secretion via specific transporters for organic anions folates, and a small part is excreted through the biliary tract. Thus, the contribution of renal secretion is greater than that of the hepatic mechanism [32, 37]. Slow elimination of MTX in patients with renal insufficiency leads to prolonged exposure of bone marrow tissues to this drug. Renal insufficiency has been incriminated as the major risk factor for myelosuppression [20–22, 24, 29, 30, 38, 39], and our recent real-life registered study for RA showed that approximately one-fourth of patients have renal insufficiency [26]. In the present study, corrected eGFR was significantly associated with the development of myelosuppression during low-dose MTX therapy, but it was less likely to be the significant factor associated with the severity of pancytopenia or neutropenia.

Interactions with antirheumatic drugs commonly administered with MTX, such as NSAIDs, prednisolone, and other DMARDs, may influence MTX toxicity [40]. In most previous case reports of MTX-related pancytopenia, patients had received multiple medications concomitantly [20–22, 24, 29, 38]. Excretion of MTX can be inhibited by the interaction with NSAIDs through several mechanisms, including decreased renal blood flow and glomerular filtration as well as competitive inhibition of active tubular secretion of MTX. In addition, almost all NSAIDs are more than 90% bound to plasma proteins, which can lead to competition with MTX for binding-sites on albumin [41]. Through a systemic review of studies examining concurrent use of MTX and NSAIDs, however, Colebatch et al. showed that there was no increase in the rates of MTX withdrawal or major toxic reactions in the management of RA [42]. Only one study reported a correlation between transient thrombocytopenia and concurrent use with NSAIDs [43]. There are no definitive data published regarding whether there are possible drug interactions between prednisolone and MTX [44]. Concerning other DMARDs, sulfasalazine is known to inhibit active tubular excretion of MTX via the specific transporters [45], but it is not clear whether this interaction is clinically significant in hematological toxicity. Concurrent use of MTX with leflunomide is also reported to increase the risk of pancytopenia [46]. Regarding pharmacokinetic interactions between MTX and biological DMARDs, very few data have been published [44]. In the present study, we showed that concurrent medications, especially NSAIDs, antacids, and prednisolone, were the significant factors associated with the development of myelosuppression. There is the possibility that myelosuppression may have occurred more often in rheumatic disease patients whose disease activity was not well controlled. Higher blood cell counts (leukocytes, neutrophils, and thrombocytes) and lower hemoglobin levels were seen in the myelosuppression group, which may also suggest this possibility.

One of the main limitations of the present study is its small sample size, which prevented us to draw a more definitive conclusion. Nevertheless, our cohort of 40 patients is larger than that of any of the past studies in the literature. Second, we did not know, as the denominator, the exact number of patients who had received MTX because this study included patients who had started MTX therapy in other hospitals. Therefore it was impossible to calculate a true prevalence of myelosuppression related to this therapy. Third, the present study was performed retrospectively, which may confer certain inherent limitations, such as bias and confounding, on the study. However, since pancytopenia and neutropenia are rare complications in rheumatic disease patients receiving low-dose MTX [15–23], it was difficult to perform a prospective registry study, as the registry size required was too large to be feasible.

## Conclusions

Serum albumin levels and folic acid supplementation were significantly associated with the severity of pancytopenia and neutropenia. Although low renal function and concurrent medications were the significant risk factors for the development of myelosuppression, they were less potent in affecting the disease severity. Myelosuppression occurred abruptly at any time during low-dose MTX therapy, but severe neutropenia was more often seen in the early-onset period of this therapy. Contrary to our expectations, disease severity was not dose-dependent in low-dose MTX therapy. We should keep in mind that the early detection of myelosuppression is a challenging task, even when monthly blood monitoring is adequately performed during low-dose MTX therapy.
